# Gene co-expression changes underlying the functional connectomic alterations in Alzheimer’s disease

**DOI:** 10.1186/s12920-022-01244-6

**Published:** 2022-04-23

**Authors:** Bing He, Priyanka Gorijala, Linhui Xie, Sha Cao, Jingwen Yan

**Affiliations:** 1 Department of BioHealth Informatics, Indiana University Purdue University Indianapolis, Indianapolis, IN, USA; 2 Department of Electrical and Computer Engineering, Indiana University Purdue University Indianapolis, Indianapolis, IN, USA; 3 Department of Biostatistics and Health Data Sciences, School of Medicine, Indiana University School of Medicine, Indianapolis, IN, USA

**Keywords:** Differential co-expression, Functional connectivity, Alzheimer’s disease

## Abstract

**Background:**

There is growing evidence indicating that a number of functional connectivity networks are disrupted at each stage of the full clinical Alzheimer’s disease spectrum. Such differences are also detectable in cognitive normal (CN) carrying mutations of AD risk genes, suggesting a substantial relationship between genetics and AD-altered functional brain networks. However, direct genetic effect on functional connectivity networks has not been measured.

**Methods:**

Leveraging existing AD functional connectivity studies collected in NeuroSynth, we performed a meta-analysis to identify two sets of brain regions: ones with altered functional connectivity in resting state network and ones without. Then with the brain-wide gene expression data in the Allen Human Brain Atlas, we applied a new biclustering method to identify a set of genes with differential co-expression patterns between these two set of brain regions.

**Results:**

Differential co-expression analysis using biclustering method led to a subset of 38 genes which showed distinctive co-expression patterns between AD-related and non AD-related brain regions in default mode network. More specifically, we observed 4 sub-clusters with noticeable co-expression difference, where the difference in correlations is above 0.5 on average.

**Conclusions:**

This work applies a new biclustering method to search for a subset of genes with altered co-expression patterns in AD-related default mode network regions. Compared with traditional differential expression analysis, differential co-expression analysis yielded many more significant hits with extra insights into the wiring mechanism between genes. Particularly, the differential co-expression pattern was observed between two sets of genes, suggesting potential upstream genetic regulators in AD development.

## Introduction

Human brain functions as a network of multiple brain regions. Each brain region has its own function but also shares information with each other. Therefore, they form a complex comprehensive network in which information is continuously processed and transmitted between structurally and functionally connected brain regions [1]. Functional brain connectivity is captured based on the assessment of temporal correlations between different brain regions of interest (ROIs) using functional magnetic resonance imaging (fMRI). There has been growing evidence that a number of functional connectivity networks are disrupted at each stage of the full clinical Alzheimer’s disease (AD) spectrum [2–4]. Particularly, such neural differences are also detectable in cognitive normal (CN) carrying mutations of AD risk genes, suggesting a substantial relationship between genetics and AD-altered functional brain networks [5]. However, direct genetic effect on functional connectivity networks has not been measured [5, 6].

Present genetic association studies of brain connectivity are mostly for structural brain network with a primary focus on examining pairwise univariate associations between genetic markers such as Single Nucleotide Polymorphisms (SNPs) and basic connectome measures at each edge [7] or at each voxel [8, 9]. A few recent studies worked on association brain expression with brain connectivity and allowed detection of underlying genes [4, 10]. However, only the expression and connectivity in each brain region were examined.

Recently, the Allen Human Brain Atlas (AHBA) (https://portal.brain-map.org/) provided a complete transcriptomic atlas inside the brain. It measured the expression level of more than 10,000 genes across  1000 brain samples. This data allows us to study another type of brain connectivity, i.e., co-expression network between brain regions, which captures the similarity of brain regions based on the expression level of a set of genes. Recent analyses have used the AHBA data to look for the associations between co-expression brain network and the organization of human brain functional connectivity [11–14]. However, the co-expression brain network is mostly built using all genes. This strategy might miss some signal since a certain brain function only involves a set of genes. Using all genes to study the brain inter-regional co-expression will possibly mask some pattern out. Since the human brain expression data is only limited to postmortem brains, it is difficult to meaningfully relate gene expression with functional connectivity when performing a specific task. Therefore, most of those studies focused on the functional connectivity measured under resting state, which has been previously found to be consistent across healthy subjects [15, 16].

Leveraging this brain wide gene expression data, we proposed a novel strategy to explore the transcriptomic changes underlying the functional brain connectivities altered in AD brains, including both differential expression and differential co-expression. Specifically, we first performed a meta-analysis of existing AD resting state network studies collected in NeuroSynth, and identified two sets of brain regions: ones with altered functional connectivity in resting state network and ones without. Then with the brain-wide gene expression data in the Allen Human Brain Atlas, we applied a biclustering method to identify a subset of genes with differential co-expression patterns between these two set of brain regions. In total, we were able to identify 38 genes in 4 subnetwork modules with differential co-expression patterns, which in further analysis are found closely connected to known AD risk genes.

## Methods

### Functional connectivity data

We used NeuroSynth (https://neurosynth.org/) database to collect the functional connectivity papers related to AD. It is a web platform established in 2011 for large-scale automated synthesis of functional magnetic resonance imaging (fMRI) data. To the best of our knowledge, Neurosynth is by far the largest and most up-to-date collection of functional connectivity studies. It has collected 14,371 papers on fMRI studies with reported MNI coordinates of activation sites.

In total, 137 papers in NeuroSynth are listed under the term “Alzheimer” and 60 of them are focused on resting state functional connectivity. All these resting state papers were further categorized according to the diagnosis groups used for comparison, like AD vs normal control, mild cognitive impairment (MCI) vs normal control, MCI vs AD, MCI only and Mild AD vs Normal control. Some papers included only one diagnosis group and some have more than 2 diagnosis groups. Since the meta-analysis tool in NeuroSynth does not differentiate the activation patterns between each pair of groups if more than two diagnosis groups exist, we only focused 16 studies performing comparison analysis between Alzheimer patients and normal controls.

### Meta-analysis of functional connectivity altered in AD

Meta-analysis of co-activation patterns reported in 16 pre-selected papers was performed using the core package provided by NeuroSynth, a lightweight set of Python modules that support large-scale automated synthesis and manipulation of functional MRI activation. First, each paper was labeled with multiple terms and a corresponding binary image mask was generated, with a value of 1 (reported) assigned to each voxel in the brain if it was within a focus reported in that article and 0 (not reported) if it was not. Based on that, for each brain voxel and term (e.g., task), every study can be cross-classified by activation (present or absent) and term (present or absent), producing a 2 $\times$ 2 contingency table of counts. The statistical inference was then done using chi-square test, with a significant result implying the presence of a dependency between term and activation. As a result, a Nifti file indicating the voxel level significance of co-activation was generated. In total, 6982 voxels passed the significance threshold (FDR corrected $p\le 0.05$) and were considered with altered functional connectivity in AD patients. We further mapped each of these voxels to Glasser atlas based on their MNI coordinates using Resting-State fMRI Data Analysis Toolkit [17]. Out of 6982 voxels, 6622 of them were located within 2mm distance of Glasser regions and therefore are mapped to their closest Glasser regions of interest (ROIs). The rest were excluded from the subsequent analysis. To focus our analysis to resting state network, we further mapped Glasser ROIs to Yeo 7 atlas [18] and 103 Glasser ROIs were found to be involved in default mode network, where 64 of them have significant voxels identified from meta-analysis and 39 of them do not. For those ROIs with significant voxels, we further excluded the ones with less than 10 significant voxels and 46 Glasser ROIs were retained.

### Gene expression data

Brain wide transcriptomic data were downloaded from the Allen Human Brain Atlas (AHBA) [14]. The AHBA includes genome-wide microarray-based expression covering the entire brain through systematic sampling of regional tissue, where $\sim$60,000 probes across approximately $\sim$1000 samples were collected for each individual postmortem brain. The samples are distributed across cortical, subcortical, brainstem and cerebellar regions in each brain. Expression profiles for all six health human brains have been released, including two full brains and four right hemispheres.

The AHBA transcriptome data was pre-processed following a recently published protocol [19]. More specifically, first, given that microarray data only quantifies probes that correspond to a short DNA sequence, we performed a probe-to-gene mapping using Re-Annotator [20] with Genome v19. Secondly, we filtered out the probes that does not exceed the background noise based on the intensity based filtering (IBF) provided by AHBA. Only probes that exceed the background noises in at least 50% of all brain samples across all subjects be retained. In case of multiple probes corresponding to one gene, we represent the gene with the probe that shows best expression consistency across individual brains [21]. Finally, to enable the association between gene expression and functional connectivity, we mapped AHBA brain samples to the Glasser ROIs based on their Montreal Neurological Institute (MNI) coordinates. Default Mode Network (DMN) related Glasser ROIs without gene expression data was excluded for the subsequent analysis. Ultimately, we have the expression data of 10,027 genes from 46 AD-related and 20 non AD-related DMN ROIs.

Given that the genes greatly outnumbered the Glasser ROIs to be analyzed, the gene expression is represented as a flat matrix and will likely produce biased results with biclustering methods. To address this problem, we narrowed our candidate gene list based on the large-scale GWAS summary statistics from the International Genomics of Alzheimer’s Project (IGAP) [22]. In total, 7,055,881 single nucleotide polymorphisms (SNPs) of 17,008 Alzheimer’s disease cases and 37,154 controls were included in their stage 1 GWAS analysis. In stage 2, 11,632 SNPs with $p \le 10^{-6}$ were genotyped and tested for association in an independent set of 8,572 Alzheimer’s disease cases and 11,312 controls. Finally, a meta-analysis was performed combining results from stages 1 & 2 [23]. SNPs with meta analysis $p \le 5 \times 10^{-3}$ were extracted and their corresponding genes (N=946) were used for the subsequent differential expression and differential co-expression analysis.

### Differential expression analysis

Using the gene expression data in AD-related and non AD-related DMN ROIs , we performed the traditional differential expression analysis using Limma package in R [24]. All the p-values were further adjusted using FDR method to correct for multiple comparison and the significance threshold was set at 0.05.

### Differential co-expression analysis

We applied a new biclustering method to identify subgroups of genes among the 946 genes that show differential correlation patterns between the two conditions, i.e., AD-related and non AD-related DMN ROIs. Specifically, we first estimated a differential correlation matrix for the 946 genes between the two conditions, which is assumed to be a sparse matrix. We then applied a binary matrix factorization method to identify subgroups of genes that consistently shown non-zero differential correlations with each other.

We consider a data-driven adaptive thresholding method for the estimation of the differential correlation matrix proposed in [25]. Let $\mathbf {X^{(t)}}=({\varvec{X}}_1^{(t)},...,{\varvec{X}}_p^{(t)})^{T}$ be a p-variate random vector with mean $\mu _t$, covariance matrix $\varvec{\Sigma }_{t}=\left( \sigma _{i j t}\right) _{1 \le i, j \le p}$, and correlation matrix ${\mathbf {R}}_{t}=\left( r_{i j t}\right) _{1 \le i, j \le p}$, for t=1,2. Suppose we observe $n_1$ i.i.d. random samples ${\{{\varvec{X}}_1^{(1)},...,{\varvec{X}}_{n_1}^{(1)}}\}$ from ${\varvec{X}}^{(1)}$ and $n_2$ i.i.d samples random samples ${\{{\varvec{X}}_1^{(2)},...,{\varvec{X}}_{n_2}^{(2)}}\}$ from ${\varvec{X}}^{(2)}$, and the two samples are independent. The goal is to estimate the differential correlation matrix $D=R_1-R_2$, under the assumption that D is sparse. However, neither of $R_1$, $R_2$ is known, and what we observe are the sample correlation matrices denoted as $\widehat{{\mathbf {R}}}_{t}, t=1,2$, and the sample differential correlation matrix denoted as $\widehat{{\mathbf {D}}}=\widehat{{\mathbf {R}}}_1-\widehat{{\mathbf {R}}}_2$, Here $\widehat{{\mathbf {R}}}_{t}$ is calculated from the random samples of ${\varvec{X}}^{(t)}$. A key challenge to the construction of D from $\widehat{{\mathbf {D}}}$ is the estimation of the noise levels of the individual entries in $\widehat{{\mathbf {D}}}$, as these entries are random variables themselves. A data-driven approach based on cross validation is used to individually threshold the entries of $\widehat{{\mathbf {D}}}$ with the threshold adaptive to the noise level of each entry. Basically, without loss of generality, we break down the samples in each condition into five folds respectively, four folds for calculating the training differential correlation matrix denoted $\widehat{{\mathbf {D}}}_{0}$ and one fold for calculating the testing differential correlation matrix denoted $\widehat{{\mathbf {D}}}_{1}$. For any threshold parameter $\tau$, we calculate the thresholded matrix of $\widehat{{\mathbf {D}}}_{0}$ denoted as $\widetilde{{\varvec{D}}}_{0}$ as $\widetilde{{\varvec{D}}}_{0}=\widehat{{\varvec{D}}}_{0} *\left( \left| \widehat{{\varvec{D}}}_{0}\right| >\tau \right)$. This means that all entries in $\widetilde{{\varvec{D}}}_{0}$ less than $\tau$ will be thresholded to 0. And the generalization loss associated with $\tau$ will be defined as $\left\| \widetilde{{\varvec{D}}}_{0}-\widehat{{\varvec{D}}}_{1}\right\| _{F}$, where $\Vert \cdot \Vert _{F}$ denotes the Frobenius norm. We calculate such generalization loss for a grid of $\tau$, and we repeat for 100 times for each $\tau$ to select the most optimal $\tau$ with the smallest generalization loss. Denote the thresholded sample differential correlation matrix with the most optimal threshold parameter as $\widetilde{{\varvec{D}}}$, it will then be used as an estimate for the sparse matrix ${\varvec{D}}$.

On the estimated differential correlation matrix $\widetilde{{\varvec{D}}}$, we look for those groups of genes such that within the same subgroup, genes show consistently non-zero differential correlations with each other between the two conditions. This is equivalent to look for submatrix of non-zero entries in the $\widetilde{{\varvec{D}}}$ matrix. We will apply our in-house binary matrix factorization algorithm, called MEBF, to look for such submatrix in the dichotomized $\widetilde{{\varvec{D}}}$ [26]. Basically, MEBF iteratively looks for submatrices in a binary matrix that is dense in 1.

## Results

### Differential expression

Differential expression analysis yielded 1247 genes with p value less than 0.05. After multiple correction using FDR, only 3 genes were left including *VWA3A*, *TMEM18* and *ZNF845*. *VWA3A* has been previously found associated with progressive supranuclear palsy (PSP), a degenerative neurological disorder that causes progressive impairment of balance and walking [27]. DNA methylation level of *TMEM18* was significantly correlated to the burden of neuritic amyloid plaques (NP), a key quantitative measure of Alzheimer’s disease neuropathology [28]. *ZNF845* is located within a gene network module that is up-regulated in the cerebellum region of AD brains [29, 30].

### Differential co-expression analysis

**Fig. 1:**
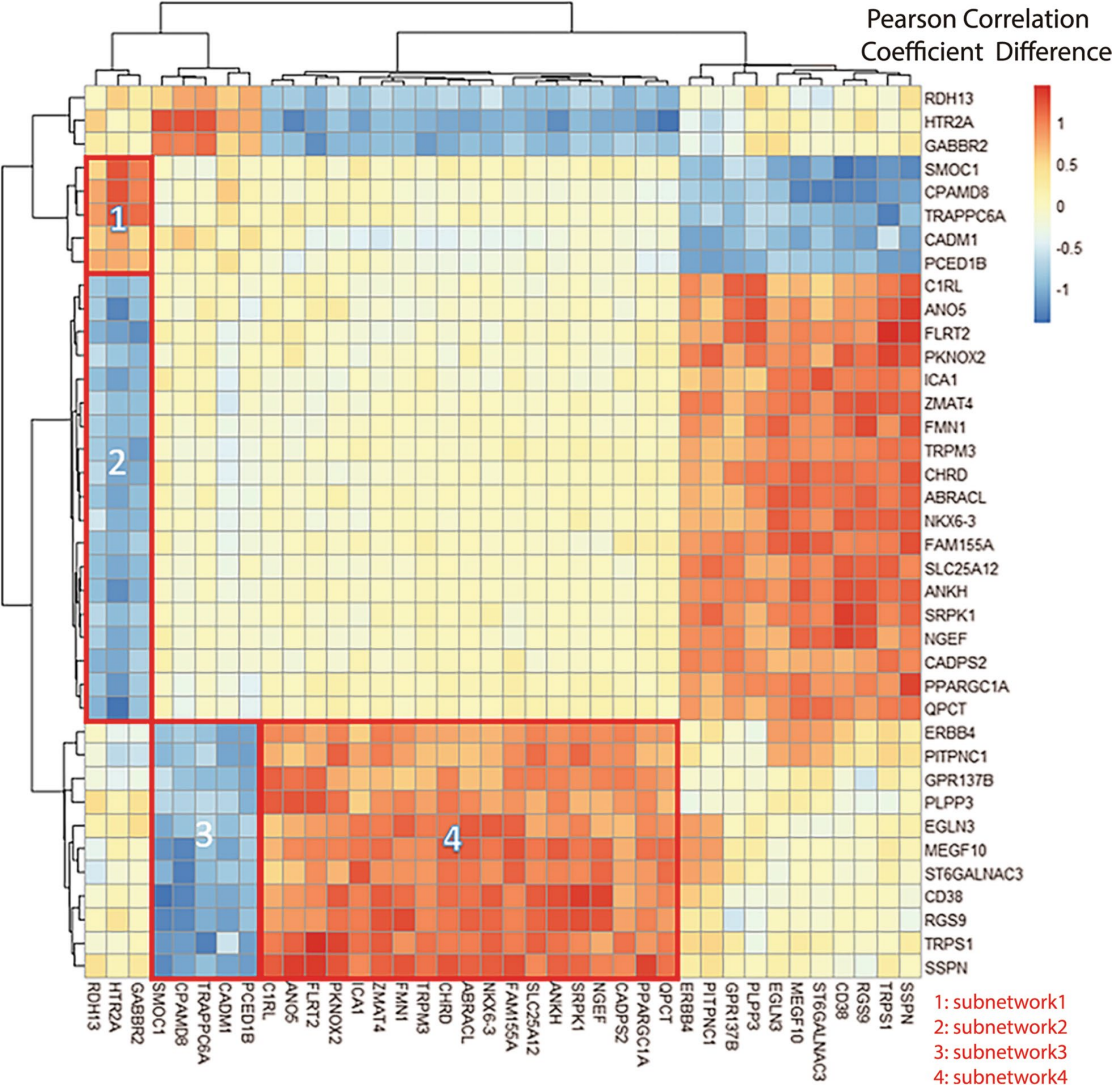
Heatmap showing difference in co-expression between a subset of genes identified from biclustering mthod

Differential co-expression analysis using biclustering method led to a subset of 38 genes which showed distinctive co-expression patterns between AD-related and non AD-related DMN ROIs (Fig. 1). More specifically, we observed 4 sub-clusters with noticeable co-expression difference, where the difference in correlations is above 0.5 on average. Overall, sub-clusters 1 and 4 have higher co-expression pattern in AD-related ROIs, and sub-clusters 2 and 3 have higher co-expression pattern in the non AD-related regions. Interestingly, these sub-clusters are all between two sets of genes without any overlap, which is very likely due to the alteration of upstream regulators.

### Pathways enrichment analysis

**Fig. 2:**
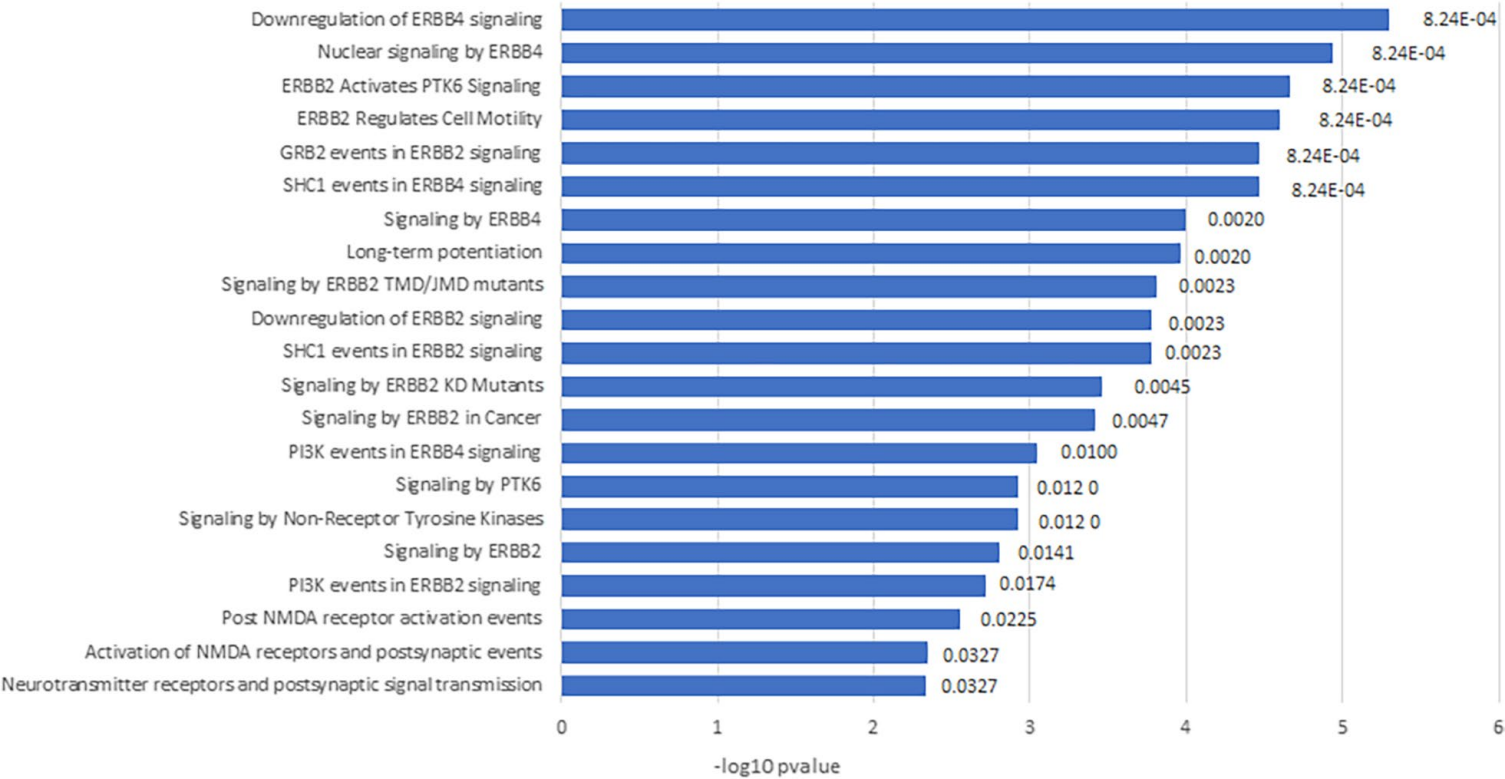
Pathways enriched by 38 differentially co-expressed genes ranked by -$log_{10}(p)$. Shown on the right of each bar is FDR corrected p-value

For 38 genes identified with differential co-expression patterns, we further performed pathway enrichment analysis to investigate potential system level perturbations. Enrichment analysis was performed using Reactome webserver (https://reactome.org/) based on pathways in REACTOME database [31]. After FDR correction, 21 pathways were found to be significantly enriched by our gene set with adjusted p-value $p \le 0.05$ (Fig. 2). Among those, top hits are mostly signaling pathways related to *ERBB4*, *ERBB2* and *PTK6*. *ERBB4* are found highly enriched in neuronal plaques of AD patients and therefore is speculated to play a role in the pathology of Alzheimer’s disease (AD) [32, 33]. Similarly, significantly high level of *ERBB2* was confirmed in the hippocampus of human AD brains. As oncogenic receptor tyrosine kinase, *ERBB2* was identified to have a critical function in its monomeric form and increased levels of *ERBB2* in the hippocampus was suggested as a potential diagnostic marker of sporadic AD [34]. Signaling by *PTK6* is activated downstream of *ERBB2*, and thus is no surprise to be identified. Another pathway that made the top hits is Long-term potentiation (LTP), whose mechanism is affected by the amyloid-$\beta$ fragments, one of the two hallmarks of AD [35]. It is a rapid and persistent increase in synaptic transmission and AD-diseased synapses are found intrinsically defective in LTP [36].

### Functional interactions with AD genes

**Fig. 3:**
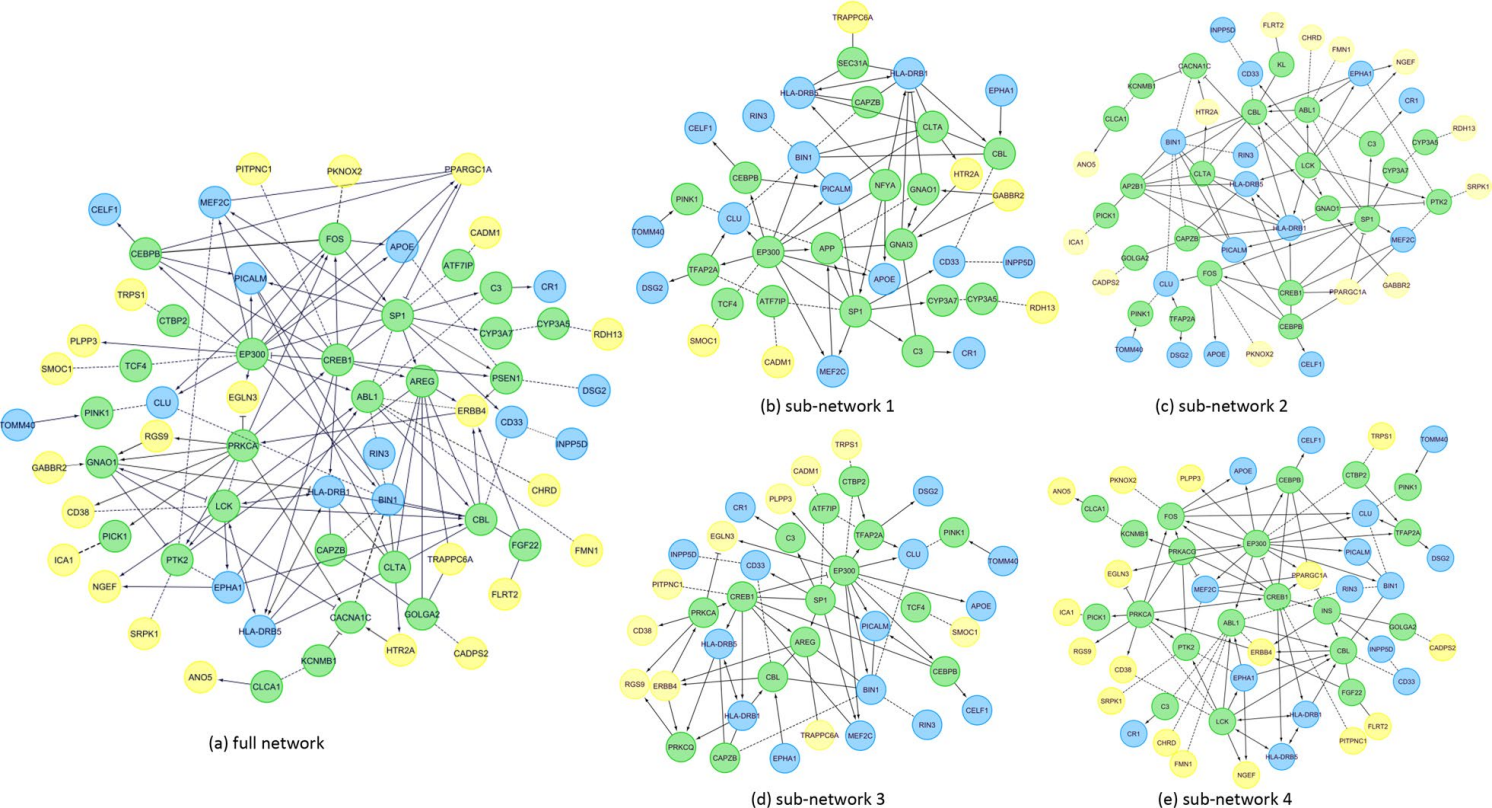
Functional interaction network between differentially co-expressed genes and AD genes. Green nodes: linker genes, Blue nodes: AD genes, Yellow nodes: differentially co-expressed genes identified from bilcustering. **a** functional interaction network generated using all differentially co-expressed genes, **b**–**f** functional interaction networks generated using differentially co-expressed genes from each sub-cluster

We further performed gene set enrichment analysis in ReactomeFI ( a Cytoscape plugin) to examine the relationship between 38 differentially co-expressed genes and known AD genes identified from large-scale GWAS [23]. In total, 15 AD genes were included, i.e., *APOE*, *TOMM40*, *CR1*, *DSG2*, *CD33*, *CLU*, *CELF*, *BIN1*, *RIN3*, *PICALM*, *EPHA1*, *INPP5D*, *MEF2C*, *HLA-DRB5*, and *HLA-DRB1*. Linker genes were used in case that two set of genes are not directly connected. As shown in Fig. 3a, 23 out of 38 genes were found to interact with others; the rest 15 genes do not connect with any other genes and thus are not shown in the network. It is observed that most differentially co-expressed genes are located downstream in the network, without direct interactions between them. This adds weights to our earlier speculation that difference in co-expression observed between two set of genes are likely due to the alteration of upstream regulators. Inside the network, we found 2 differentially co-expressed genes with high degree, *PPARG1A* and *ERBB4*. While *ERBB4* has been previously discussed, *PPARGC1A* helps decrease the generation of A$\beta$ and its levels are reduced in AD brains [37]. With all genes considered, top hub genes include *EP300*, *PRKC1*, *CREB1*, *BIN1* and *CBL*. Same gene set analysis was also performed for genes within each sub-cluster (Fig. 3b–e) and similar patterns were observed for all of them.

## Discussion

**Fig. 4:**
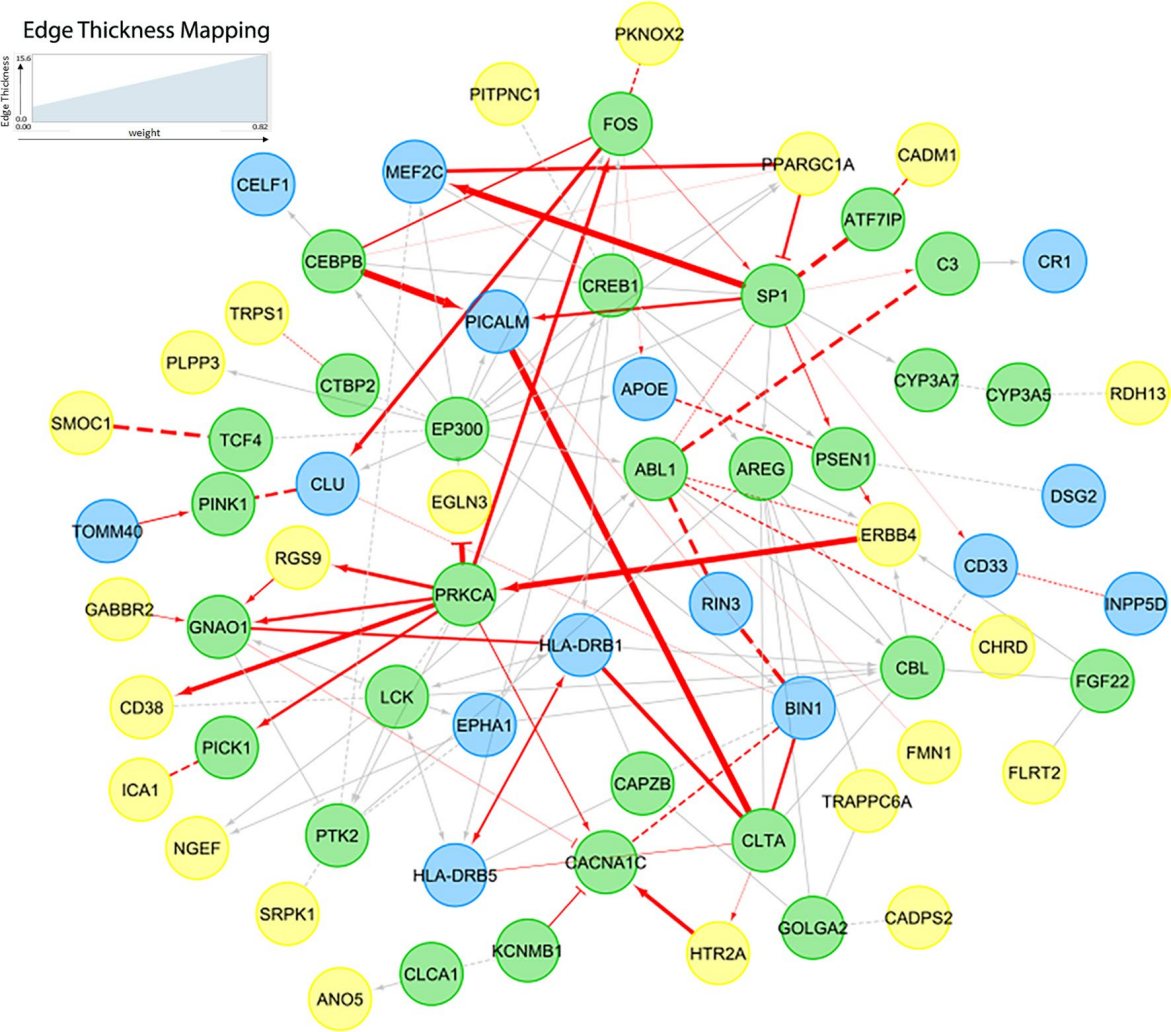
Re-evaluated difference in co-expression for all gene pairs in functional interaction network Fig. 3a. Green nodes: linker genes, Blue nodes: AD genes, Yellow nodes: differentially co-expressed genes identified from bilcustering. Gray edges are the gene pairs with missing expression data. Red color edges are genes pairs with expression data and their thickness are proportional to difference in co-expression

Most of our findings are downstream genes without direct interaction, which is likely due to that our targeted differential co-expression missed some important upstream genes. Therefore, we mapped all the genes in the subnetwork Fig. 3a to our complete brain expression data and further examined the differential co-expression patterns between gene pairs in the network. Out of 66 genes in the network, 50 of them were found to have expression level information in the processed AHBA data, where 16 of them were not examined in our targeted analysis. For all 54 gene pairs with available expression data, we re-evaluated their difference in co-expression by comparing the Pearson’s correlation between AD-related and non AD-related DMN ROIs. Shown in Fig. 4 is the differential co-expression mapped to the subnetwork in Fig. 3a. For those 54 gene pairs, edge thickness was made proportional to the difference in co-expression. The rest of edges were marked as gray due to the lack of expression data in either or both connected genes. As expected, we observed differential co-expression patterns in some upstream genes, which has not been previously included in our biclustering analysis. Particularly, genes *PRKCA*, *FOS*, *SP1*, *PICALM* and *CLTA* were found as top hubs with altered co-expression with several other genes in AD-related DMN ROIs. While *PICALM* is already a AD gene, other hub genes are also known to be closely related to AD. The one with most links as differentially co-expressed is *PRKCA* gene, which has been previously associated with an altered amyloid precursor protein (APP) secretion in fibroblasts in AD patients [38]. Three variants in *PRKCA* has been linked with increased catalytic activity displayed in late onset AD [39]. Another hub gene with many differentially co-expressed links is *CLTA*, which encodes for protein Clathrin light chain A - a potential regulator of synaptic vesicle formation. A recent co-expression analysis suggested a potential role of *CLTA* in maintaining the homeostasis of the metastable subproteome associated with Alzheimer’s disease [40]. *SP1* gene is a regulator which mediates the expression of several AD-related proteins, including amyloid precursor protein (*APP*) and tau [41].

## Conclusion

Leveraging the brain-wide gene expression data, we applied a new biclustering method to search for a subset of genes with altered co-expression patterns in AD-related default mode network regions. Compared with traditional differential expression analysis, differential co-expression analysis yielded many more significant hits with extra insights into the wiring mechanism between genes. Particularly, the differential co-expression pattern was observed between two sets of genes, suggesting some potential upstream regulators. This hypothesis was further supported by our findings that these genes were mostly located downstream of AD genes or other linker genes when mapped to functional interaction network. Considering that biclustering analysis was only applied to a set of targeted genes, we re-evaluated the difference of co-expression for all gene pairs in the enriched functional network. As expected, we observed some upstream hubs including *PICALM*, *PRKCA* and *CLTA* with altered co-expression with several other genes. All of these genes and enriched pathways are related to synaptic transmission and synapse formation, which suggests their potential role in mediating the alterations of resting state functional connectivity in AD brains.

## Data Availability

The data that support the findings of this study are available from the Allen Human Atlas (https://portal.brain-map.org/) and the NeuroSynth database (https://neurosynth.org/)

## Abbreviations

AD: Alzheimer’s disease
CN: Cognitive normal
MCI: Mild cognitive impairment
ROIs: Regions of interest
SNPs: Single nucleotide polymorphisms
fMRI: Functional Magnetic Resonance Imaging
AHBA: Allen human brain atlas
IBF: Intensity based filtering
DMN: Default mode network
MNI: Montreal neurological institute
IGAP: International genomics of Alzheimer’s project
PSP: Progressive supranuclear palsy
NP: Neuritic amyloid plaques
LTP: Long-term potentiation
APP: Amyloid precursor protein
